# Ammonium quantification in human plasma by proton nuclear magnetic resonance for staging of liver fibrosis in alcohol‐related liver disease and nonalcoholic fatty liver disease

**DOI:** 10.1002/nbm.4745

**Published:** 2022-05-09

**Authors:** Marc Azagra, Elisa Pose, Francesco De Chiara, Martina Perez, Emma Avitabile, Joan‐Marc Servitja, Laura Brugnara, Javier Ramon‐Azcón, Irene Marco‐Rius

**Affiliations:** ^1^ Institute for Bioengineering of Catalonia The Barcelona Institute of Science and Technology Barcelona Spain; ^2^ Liver Unit, Hospital Clinic, Faculty of Medicine and Health Sciences University of Barcelona Barcelona Spain; ^3^ Institut d'Investigacions Biomèdiques August Pi i Sunyer (IDIBAPS), Centro de Investigación Biomédica en Red de Diabetes y Enfermedades Metabólicas Asociadas (CIBERDEM) Barcelona Spain; ^4^ ICREA‐Institució Catalana de Recerca i Estudis Avançats Barcelona Spain

**Keywords:** ammonium quantification, blood biomarkers, chronic liver disease, disease biomarkers, hepatic dysfunction, NMR

## Abstract

Liver fibrosis staging is a key element driving the prognosis of patients with chronic liver disease. Currently, biopsy is the only technique capable of diagnosing liver fibrosis in patients with alcohol‐related liver disease (ArLD) and nonalcoholic fatty liver disease (NAFLD) unequivocally. Noninvasive (e.g. plasma‐based) biomarker assays are attractive tools to diagnose and stage disease, yet must prove that they are reliable and sensitive to be used clinically. Here, we demonstrate proton nuclear magnetic resonance as a method to rapidly quantify the endogenous concentration of ammonium ions from human plasma extracts and show their ability to report upon early and advanced stages of ArLD and NAFLD. We show that, irrespective of the disease etiology, ammonium concentration is a more robust and informative marker of fibrosis stage than current clinically assessed blood hepatic biomarkers. Subject to validation in larger cohorts, the study indicates that the method can provide accurate and rapid staging of ArLD and NAFLD without the need for an invasive biopsy.

## Graphical abstract

Blood plasma treatment followed by ^1^H‐NMR quantification of ammonium ion concentration was developed as a protocol to stage liver fibrosis. As an example application, the method is used to study differences in blood ammonium level between stages of alcoholic and nonalcoholic liver diseases.

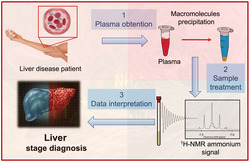

**Abbreviations used**
ALT: alanine aminotransferase
ANOVA: analysis of variance
ArLD: alcoholic‐related liver disease
ASH: alcoholic steatohepatitis
AST: aspartate transaminase
BILI: bilirubin
CHILD PUG: Child–Pugh score
DMSO: dimethylsulfoxide
DSS: sodium trimethylsilylpropanesulfonate
FWHM: full width at half maximum
GGT: gamma glutamyl transferase
INR: international normalized ratio
LOD: limit of detection
LOQ: limit of quantification
MCV: mean corpuscular volume
MELD: model for endstage liver disease
NAFLD: nonalcoholic fatty liver disease
NASH: nonalcoholic steatohepatitis
NMR: nuclear magnetic resonance
1DNOESY: selective 1D nuclear Overhauser effect spectroscopy
O1D: frequency offset
RMSE: root mean square error
RPM: revolutions per minute
SNR: signal‐to‐noise ratio
TFA: trifluoroacetic acid
T1: longitudinal relaxation

## 1 INTRODUCTION

Alcohol‐related liver disease (ArLD) and nonalcoholic fatty liver disease (NAFLD) are currently the two most frequent causes of liver disease in the Western world, accounting for the vast majority of liver‐related deaths, most of the liver transplants in Europe and North America, and associated costs.^1^, ^2^ ArLD and NAFLD progress in stages from minor fat accumulation within hepatocytes (steatosis), to liver inflammation (named alcoholic steatohepatitis [ASH] and nonalcoholic steatohepatitis [NASH] in ArLD and NAFLD, respectively), and ultimately cirrhosis and cancer, the most advanced stages of the disease when liver transplantation is required. The progression of the disease to ASH and NASH leads to the activation of hepatic stellate cells that trigger fibrogenesis pathways and cause collagen deposition in the liver.^3^ Liver fibrosis is the most important driver of progression of chronic liver disease and one of the most reliable markers of prognosis.^4^, ^5^ The current gold standard for chronic liver disease diagnosis is liver biopsy, which is invasive and may pose significant side effects.^6^ There has been a growing interest in developing noninvasive and clinically acceptable methods to assess the presence of liver fibrosis in patients with ArLD and NAFLD, of which transient elastography and serological tests have been the most promising.^7^, ^8^ However, the clinical need for an alternative to biopsy for accurate, sensitive, and noninvasive liver fibrosis diagnosis and staging in patients with ArLD and NAFLD remains unmet.

We have developed a method to quantify endogenous ammonium in blood plasma and shown it to be a sensitive biomarker of liver disease. The ammonium cation (NH_4_^+^) is an essential biomolecule in blood derived from the breakdown of nucleic acids and amino acids: the two processes used by vertebrates to fulfill their nitrogen demand.^9^ Ammonium homeostasis is governed systemically by the interplay of multiple organs including the liver, the gastrointestinal system, the muscles, the kidneys, and the brain.^10^

The physiological concentration of NH_4_^+^ in blood at equilibrium is about 40 μM in healthy subjects but may be significantly altered due to pathological conditions. For example, liver disfunction may disrupt the metabolism of NH_4_^+^ into urea and lead to a life‐threatening increase of ammonium in blood.^11^ High blood NH_4_^+^ concentration (above 50 μM) is associated with endstage liver disease, hepatic encephalopathy, neutrophil dysfunction, muscle breakdown, and cancer, although its origin remains elusive.^12^, ^13^, ^14^ In recent research, high levels of NH_4_^+^ have also been found in patients with NASH.^15^ Therefore, NH_4_^+^ levels could be a valuable biomarker of liver impairment that together with other assays could shine light on its cause. Yet, a robust and reliable method to quantify blood ammonium concentrations for samples from healthy and diseased patients is not available. Current plasma ammonia quantification methods include titration, colorimetry or fluorometry, electrode‐based, and enzymatic processes.^11^ These methods suffer from significant limitations, namely (i) sample contamination (e.g. with volatile bases during titration), (ii) interference from other compounds normally present in blood,^16^ (iii) suboptimal environmental conditions for the proper activity of the enzymes involved in the measurement (e.g. low pH and the presence of inhibitors in severe liver disease),^17^ and (iv) method calibration using samples of healthy subjects. The latter is critical in a clinical setting, as healthy individuals represent a relatively homogenous sample pool with well‐known basal conditions, while samples from patients with various stages of liver disease present heterogeneous conditions that may affect the NH_4_^+^ readout (e.g. blood pH or lipids concentration). Furthermore, certain biochemical processes such as deamination in patients with elevated gamma glutamyl transferase (GGT) activity and as a consequence of haemolysis can alter blood NH_4_^+^ concentrations postextraction, confounding quantification.^18^

Proton nuclear magnetic resonance (^1^H‐NMR) is a reliable quantitative analytical tool for small molecules that potentially provides the sensitivity and specificity needed to measure endogenous ammonia in blood. While the characteristic equi‐intense triplet of NH_4_^+^ has been detected by ^1^H‐NMR in a wide range of systems (e.g. electrochemical and photochemical N_2_ reduction, electrocatalytic N_2_ reduction, and chemical reaction solution),^19^, ^20^ measurement of endogenous NH_4_^+^ levels has remained elusive in solutions of biological origin. To detect and quantify ammonium accurately in plasma using NMR, we have developed a tightly controlled protocol to process the samples containing the analyte. The study systematically assesses factors influencing accurate NH_4_^+^ quantification of ammonia in plasma samples by NMR including solvent deuteration, pH, and NMR acquisition parameters, to determine the limit of detection (LOD) and limit of quantification (LOQ). The protocol established enables reproducible quantification of ammonium in plasma for concentrations above 3 μM. The potential for clinical application of this method is assessed by comparing the correlations between ammonium concentrations and other commonly used clinical biomarkers of liver disease. These comparisons showed that the strong correlation between ammonium concentration determined by ^1^H‐NMR readout and disease progression is likely to be a better diagnostic marker of liver disease stage than current blood hepatic biomarkers, regardless of disease etiology.

In summary, the data demonstrate that ammonium can be accurately and consistently measured in human blood plasma using a simple protocol. Validation in studies of samples from larger cohorts would simplify the way liver pathologies are diagnosed by reducing the need for biopsies with their associated risks.

## 2 EXPERIMENTAL

### 2.1 Chemicals

Chemicals were purchased from Sigma‐Aldrich (Haverhill, UK) unless stated otherwise.

### 2.2 Solvent and pH optimization

Aqueous solutions were prepared by dissolving ammonium chloride (NH_4_Cl, 107.0 mg) in H_2_O (200 ml). This solution was acidified to pH 5.5 using 1 M HCl in water. A 1‐ml aliquot was extracted, and the remaining solution was further acidified with HCl until pH 4.5. The same procedure was repeated for pHs of 4.0, 3.5, 3.0, 2.5, 2.0, 1.5, and 1.0.

Dimethylsulfoxide (DMSO) solutions were prepared by mixing 1 ml of DMSO with trifluoroacetic acid (TFA) added to give TFA concentrations of 0.15, 0.75, 1.3, and 2.6 M; 1 ml of each solution was used to dissolve NH_4_Cl (5.0 mg) in each vial. All samples were diluted with 100 ml of DMSO‐d_6_. The displacement of the water/TFA chemical shift in the ^1^H‐NMR indicated the pH changes upon addition of TFA.

### 2.3 Calibration curve of ammonium chloride in DMSO‐d_6_

A stock solution of 1 M NH_4_Cl in DMSO (268.3 mg in 5 ml) was prepared and diluted with DMSO to give final NH_4_^+^ concentrations of 26.9, 16.8, 6.7, 3.4, and 0.3 μM. All samples were acidified with 2.6 M of TFA.

### 2.4 Human plasma obtention

Written informed consent was obtained from all subjects prior to participation. The experimental protocol was approved by the research and ethics committees of the hospitals where the study was carried out, in accordance with the Declaration of Helsinki (project number HCB/2015/0653). Plasma samples were collected from five healthy subjects, 10 patients with NAFLD, and 10 patients with ArLD (aged 26–73 years). Patients were classified based on the stage of liver fibrosis observed in the liver biopsy into either nonsignificant/early‐stage fibrosis (F0‐F1 stages) or significant/advanced‐stage liver fibrosis (F2‐F4 stages) based on the Metavir scale.^21^ For both NAFLD and ArLD groups, five patients had early‐stage fibrosis and five patients presented advanced fibrosis. Blood samples were obtained from the antecubital vein after a minimum of 10 min of rest. The blood was centrifugated in a refrigerated centrifuge for 10 min at 3500 rpm. Plasma aliquots were stored at 193 K.

### 2.5 Human plasma clinical biomarkers

Routine liver function tests, biochemistry, hematology, and coagulation assessments were performed on all samples from patients with NAFLD and ArLD.

### 2.6 Human plasma sample preparation for ammonium assay by ^1^H‐NMR

Each plasma sample (0.4–0.6 ml) was transferred into a 2‐ml conical flask and treated with TFA (200 μl) to acidify the solution. The sample was snap‐frozen in liquid nitrogen and lyophilized for at least 8 h. The residue was dissolved in a solution containing TFA (200 μl), DMSO (300 μl) and DMSO‐d_6_ (300 μl), and centrifuged (10,000 rpm, 5 min). The liquid phase was removed from the conical flask and the whole sample (800 μl) was transferred into a 5‐mm OD NMR tube.

### 2.7 Ammonium quantification by ^1^H‐NMR

NMR spectra of ammonium in aqueous solutions were acquired at 300 K using a 14.1‐T Bruker Avance‐II + spectrometer. Subsequent NMR experiments in DMSO were performed at 298 K and 9.4 T in a Bruker Avance‐III HD spectrometer equipped with a cryoprobe and TopSpin 2.1 software. For ^1^H chemical shifts reference and ammonium quantification, 10 μl of a standard of sodium trimethylsilylpropanesulfonate (DSS; Cortecnet, Paris, France) in water were added to each sample tube (δ_DSS_ = 0.000 ppm; final DSS concentration = 0.14 mM).

The longitudinal relaxation time constant (T_1_) of both DSS and NH_4_^+^ in DMSO was measured in triplicate using an inversion recovery pulse sequence. Because DSS had the longest T_1_ (T_1,DSS_ = 1.6 ± 0.1 s; T_1,NH4_ = 0.5 ± 0.1 s), the relaxation delay time (d_1_) of subsequent quantitative NMR experiments was set to more than five times T_1,DSS_. The acquisition parameters for all ^1^H‐NMR measurements were an acquisition time of 2.5 s; 64 K data points; 90° flip angle; d_1_ = 15 s; and 128 scans. Solvent suppression experiments were performed with a 1D NOESY presaturation pulse sequence with the above acquisition parameters and the offset presaturation frequency at 4.71 ppm.

### 2.8 Ammonium chloride standards quantification by ^1^H‐NMR

Next, 2.2 mg of ammonium chloride were dissolved into 10 ml of water to obtain a stock solution of 4.1 mM. Then, 10 μl of this stock solution were diluted with 5 ml of water to obtain a standard 80 μM solution. Two replicates with 300 μl of this solution were transferred into conical flasks, and then 100 μl of TFA were added into each aliquote. Both samples were lyophilized overnight. The samples were redissolved with 600 μl of a solution containing TFA, DMSO, and DMSO‐d_6_ (2:3:3) immediately prior to ^1^H‐NMR acquisition and the corresponding quantifications of the ammonium concentration. The error associated with each value was calculated with propagation of uncertainty.

### 2.9 Data analysis

Data were processed using MestReNova software (Mestrelab Research, v. 14.2.0). NMR signals were zero‐filled from 16 K to 64 K, Fourier‐transformed, and then baseline‐corrected using a third‐order polynomial fit. The signal‐to‐noise ratio (SNR) of the ammonium signal was calculated as the ratio of the amplitude of each of the ammonium peaks divided by the root‐mean‐square deviation of the noise. The full width at half maximum (FWHM) value for each peak of the ammonium triplet was determined by measuring the width of the peak at half of its maximum intensity. SNR and FWHM are reported as the mean ± standard deviation of the three ammonium peaks.

Ammonium concentration in micromoles per liter ( μM) was quantified using the formula:

$$\left[{{\mathrm{NH}}_{4}}^{+}\right]\;\left(\mathrm{\mu M}\right)=\frac{n_{\mathrm{DSS}}I_{{\mathrm{NH}}_{4}^{+}}H_{\mathrm{DSS}}10^{6}}{I_{\mathrm{DSS}}H_{{\mathrm{NH}}_{4}^{+}}V_{\text{plasma}}},$$

where n_DSS_ is the number of moles of the DSS standard, I_NH4+_ is the integral of the ammonium peak, I_DSS_ is the integral of the DSS peak, H_DSS_ is the number of protons per DSS molecule that give rise to the integrated DSS peak, H_NH4+_ is the number of protons that give rise to the integrated ammonium triplet, and V_plasma_ is entered as the total volume of the initial plasma sample in liters.

As suggested by Sun et al.,^22^ the LOD, defined as the lowest analyte concentration likely to be reliably distinguished from the noise, was set at SNR = 3. The LOQ, where the lowest concentration of an analyte can be reliably assayed, was set at SNR = 10.^22^

### 2.10 Statistical analysis

Continuous variables in blood biomarkers were analysed using ANOVA test; when significant, post hoc tests were performed among groups using the Mann–Whitney test. Spearman nonparametric correlation test with two‐tailed *p*‐value was used to evaluate the link between ammonium and the other measured blood parameters. All the analyses were performed using GraphPad Software (San Diego, CA, USA).

## 3 RESULTS

### 3.1 Ammonium quantification

**FIGURE 1:**
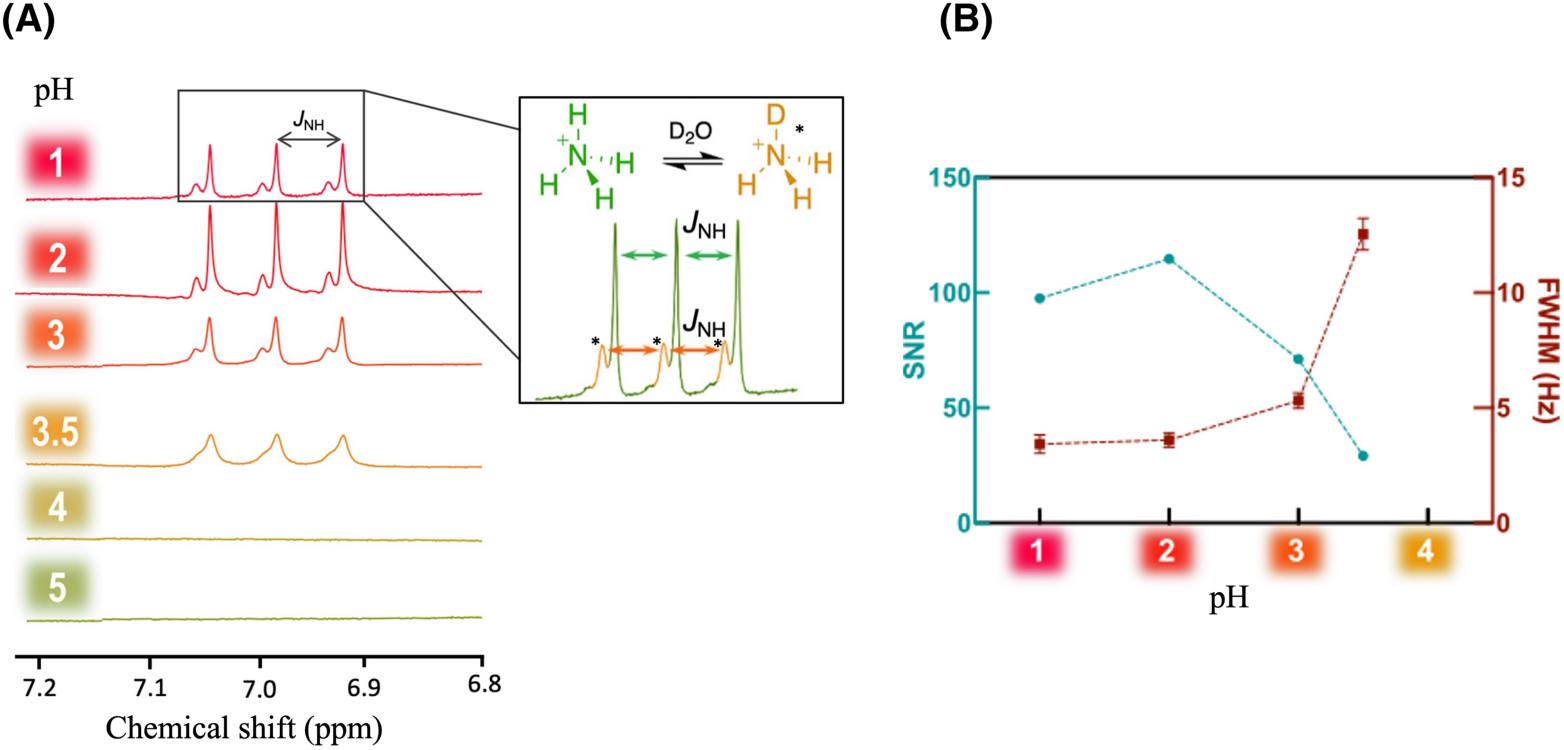
Nuclear magnetic resonance (NMR) spectra dataset of 10 mM ammonium chloride in aqueous solution (9:1 H_2_O/D_2_O). (A) ^1^H‐NMR spectra as a function of pH (chemical shifts and signal intensity referenced to the disodium trimethylsilyl propanesulfonate (DSS) peak). The concentration of ammonium in the solution at pH 1 was about 10% lower than in the other solutions caused by the dilution of the sample during acidification, which accounts for the signal drop. NMR acquisition parameters: 32 scans; 15 s relaxation delay time; 8.5 μs pulse width. (B) Signal‐to‐noise ratio (SNR) (green circles) and full width at half maximum (FWHM) (red squares) of the ammonium signal as a function of pH shown in (A). Ammonium was undetectable at pH > 3.5

**FIGURE 2:**
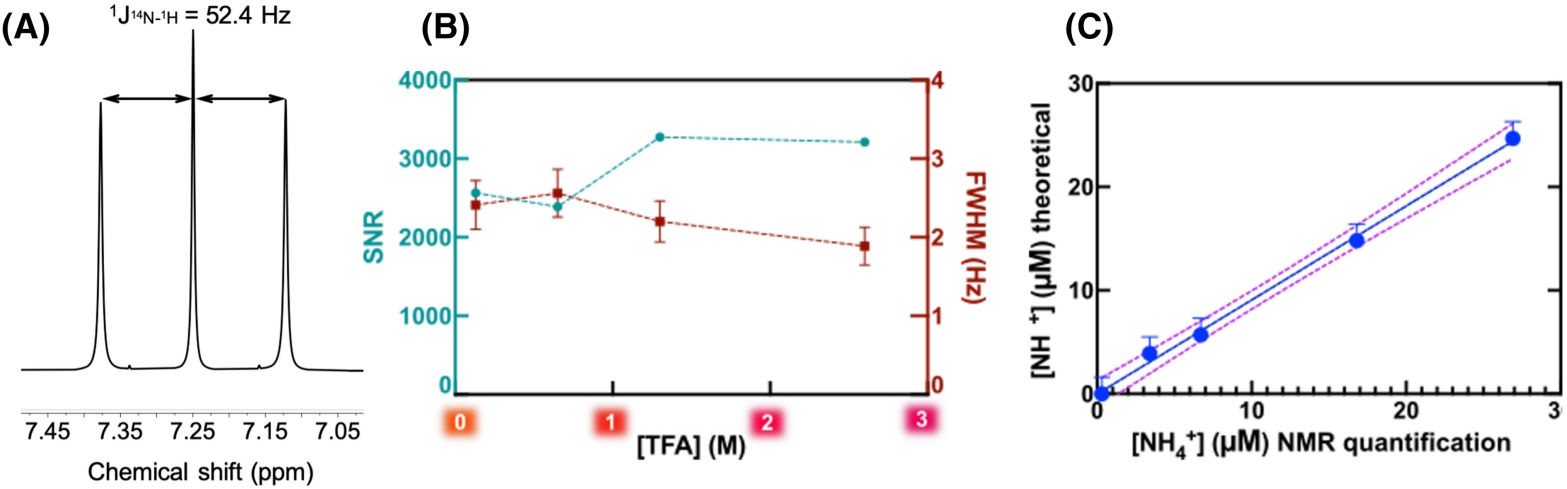
Ammonium chloride in dimethyl sulfoxide and deuterated dimethyl sulfoxide (1:1 DMSO/DMSO‐d_6_). (A) Proton nuclear magnetic resonance (^1^H‐NMR) spectrum (chemical shifts referenced to the DSS peak). NMR acquisition parameters: 32 scans; 15 s relaxation delay time; 8.5 μs pulse width. (B) Signal‐to‐noise ratio (SNR) and full width at half maximum (FWHM) as a function of final trifluoroacetic acid (TFA) concentration. Although pH measurement in organic solvents is problematic, the acidity increases with TFA concentration. (C) [NH_4_^+^] calibration curve with 2.6 M TFA added to the DMSO solution. DSS, sodium trimethylsilylpropanesulfonate

We investigated the effect of pH and two solvents (9:1 water/deuterium oxide [D_2_O] and 1:1 dimethyl sulfoxide [DMSO]/DMSO‐d_6_) on the detection of the ammonium ^1^H‐NMR signal and on quantification in solutions of 10 mM ammonium. The ammonium ion contains one nitrogen atom and four chemically equivalent hydrogens with a heteronuclear J‐coupling, J_NH_, of 52.4 Hz. The pKa of the NH_3_/NH_4_^+^ pair is 9.2. Therefore NH_4_^+^ is the dominant form in the acidic solutions (pH < 6) examined. The characteristic equi‐intense triplet with the J_NH_ splitting of the ^1^H atoms of ammonium was centered at 7.00 ppm in water (Figure 1A) and at 7.25 ppm in DMSO (Figure 2A).

#### 3.1.1 In aqueous solution

In the aqueous solution, the addition of 10% D_2_O for locking caused a splitting of the ammonium signal into a lower‐intensity triplet with the same J_NH_ splitting (Figure 1A) centered at 7.00 ppm.

The chemical shift of the ammonium triplet was independent of sample pH, but peaks narrowed, and the SNR was highest in acidic conditions, with an upper threshold at pH 2 or less (Figure 1B). Ammonium quantification was accurate within 95% for pH values below pH 2 (Table **S1**). The ammonium signal was undetectable for solutions above pH 3.5 (Figure 1A).

Because the protons in the ammonium ion are in rapid exchange with the protons in the solvent, presaturation of the water peak caused a decrease in the ammonium signal by a factor of 100 (Table S2). Consequently, ammonium quantification was underestimated by more than 25‐fold when water presaturation was applied (Table S2). Without presaturation the large water peak poses a dynamic range problem for the detection of submolar concentrations of ammonium, causing baseline distortion and decreased ammonium SNR. Aqueous solvents are not, therefore, suitable for ammonium measurement.

#### 3.1.2 In nonaqueous solution

An organic solvent (DMSO/DMSO‐d_6_) was therefore used to dissolve ammonium chloride and its suitability for the detection and quantification of ammonium was determined. To avoid the problem of determining pH in organic solvents, the samples in DMSO were acidified with TFA, which has no proton signal, at final TFA concentrations of 0.13–2.6 M. The SNR of the NH_4_^+^ signal was highest at TFA concentrations of more than 1.3 M (Figure 2B, Table S3).

The calibration curve of ammonium chloride in DMSO/DMSO‐d_6_ (0.3–27 μM NH_4_Cl and 2.6 M TFA) using ^1^H‐NMR without presaturation was linear with a root mean square error (RMSE) of 1.6 (Figure 2C), a LOD of ~3 μM, and a LOQ of ~5 μM.

To measure ammonium in aqueous biological samples with a large initial water content, a lyophilization procedure that preserved the amount of ammonium present was required. To confirm that the concentration of ammonium was not affected by the lyophilization process, two standard solutions of ammonium chloride in water were prepared to a final concentration of 40 μM and were treated by same procedure as the biological samples. The ammonium chloride samples were lyophilized, redissolved in a solution of DMSO and TFA, and their ^1^H‐NMR signal measured. The corresponding ammonium quantifications by ^1^H‐NMR after the lyophilization step were 40.2 ± 0.2 and 40.5 ± 0.2 μM, respectively. The concentration of ammonium was not determined by NMR before the lyophilization step because the sample was in aqueous solution. As explained above, the partial deuteration, water signal overlap, and presaturation effects may result in unreliable measurements of ammonium concentrations.

### 3.2 Ammonium quantification in human plasma

In the optimized protocol, each plasma sample was transferred into a 2‐ml conical flask and treated with 200 μl of TFA to acidify the solution. The sample was snap‐frozen in liquid nitrogen and lyophilized for at least 8 h. The resulting powder was redissolved in 0.8 ml of a solution containing TFA, DMSO, and DMSO‐d_6_ (2:3:3)_._ Adding TFA to the samples before the lyophilization step was essential to detect an ammonium signal at the end of the process.

**FIGURE 3:**
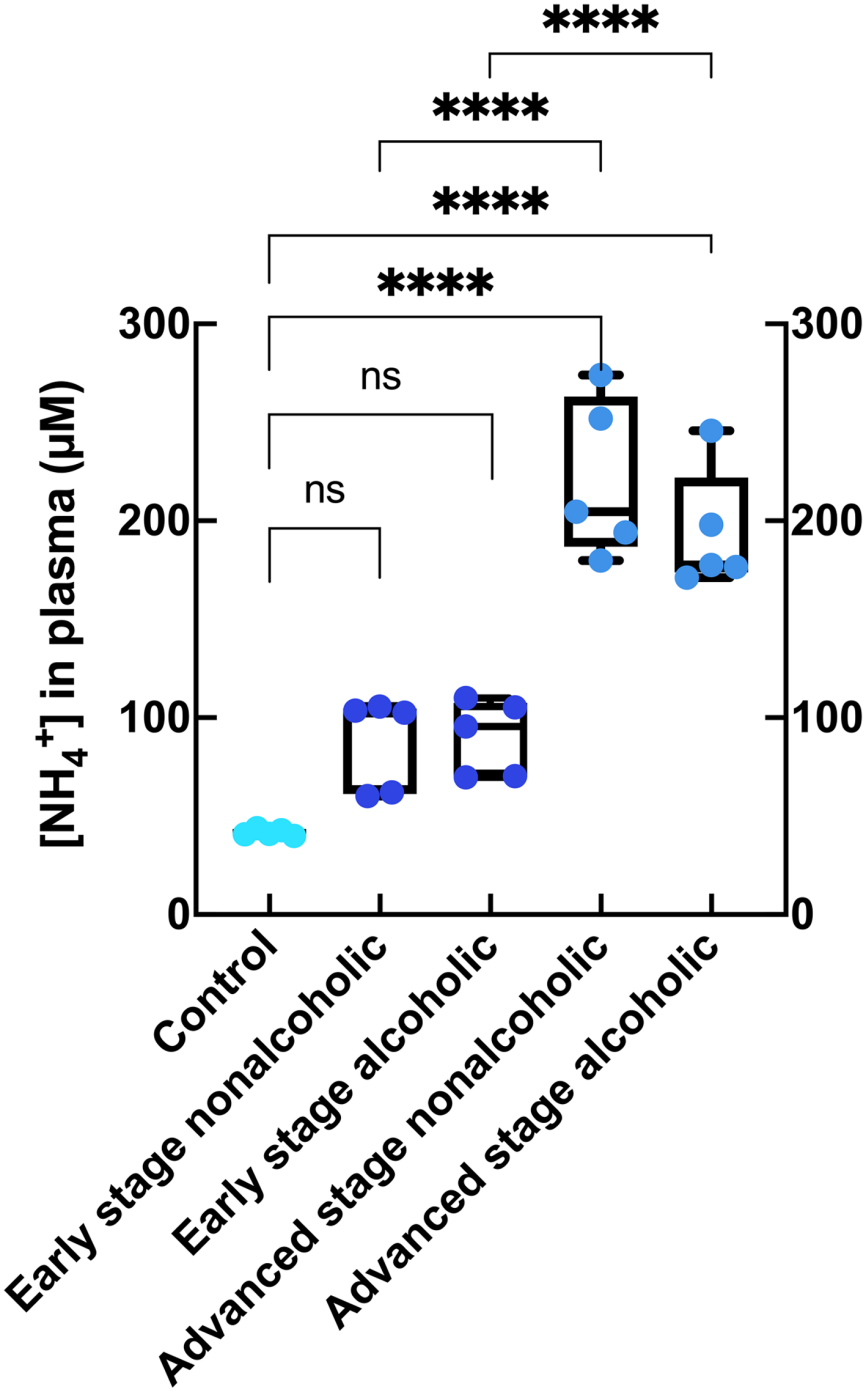
Concentration of ammonium present in plasma from healthy subjects (control; n = 5), initial and advanced stages of alcohol‐related fatty liver disease (n = 5 for each group), and initial and advanced stages of nonalcoholic fatty liver disease (n = 5 for each group); **, *p* < 0.01; ***, *p* < 0.001; ****, *p* < 0.0001

**FIGURE 4:**
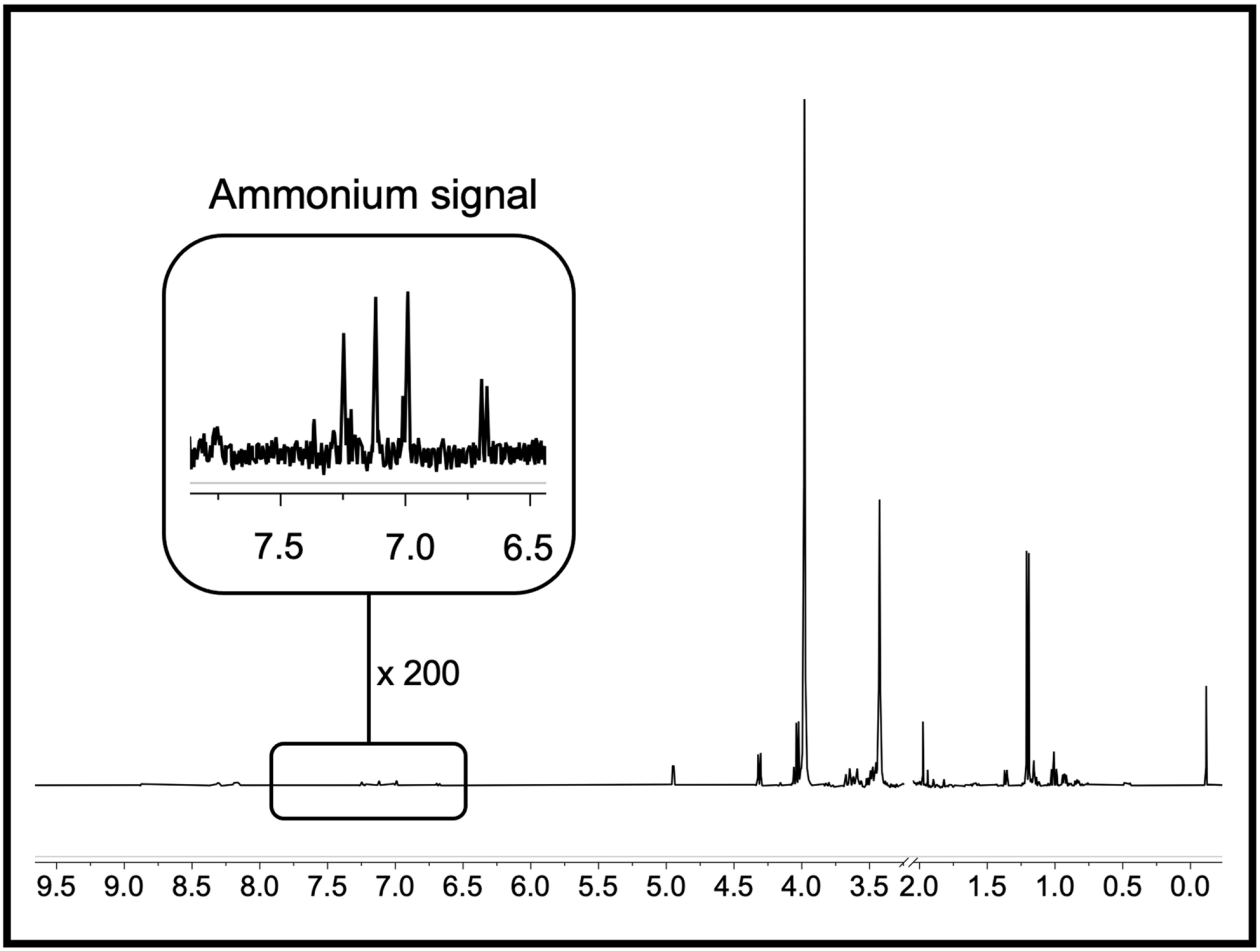
Representative proton nuclear magnetic resonance (^1^H‐NMR) spectrum of blood plasma from an advanced‐stage nonalcoholic fatty liver disease patient after lyophilization and dissolving in dimethyl sulfoxide (1:1 DMSO/DMSO‐d_6_). Chemical shifts are referenced to DSS. NMR acquisition parameters: 128 scans; 15 s relaxation delay time; 8.5 μs rf pulse width. DSS, sodium trimethylsilylpropanesulfonate

The resulting ammonium concentrations obtained from the samples extracted from healthy subjects (control group) and subjects with initial and advanced stages of either ArLD or NAFLD are shown in Figure 3. Figure 4 displays a representative ^1^H‐spectrum used for the ammonium quantification. The plasma ammonium concentration of healthy subjects was 41.76 ± 1.64 μM. Plasma ammonium concentrations of patients with initial stages of liver disease were not significantly different than those of control samples, regardless of etiology (*p* = 0.085 for control vs. alcoholic, and *p* = 0.13 for control vs. nonalcoholic). Plasma ammonium concentration of patients with advanced stages of disease was approximately five times higher than the controls (*p* < 0.0001 for both) (Table 1). There was no significant difference between groups with alcoholic and nonalcoholic initial stages of the disease.

**TABLE 1:** Clinical and laboratorial data of patients with different stages of nonalcoholic fatty liver disease (NAFLD) or alcohol‐related liver disease (ArLD). Biochemical values were obtained using standard blood analysis from a cohort of patients with either initial or advanced stages of NAFLD and another cohort of patients with ArLD (n = 5 patients per group). Significant differences in the biochemical values between initial and advanced stages of NAFLD or ArLD are highlighted in bold (*p* ≤ 0.05 calculated using nonparametric T‐test)

	IS‐NAFLD	AS‐NAFLD		IS‐ArLD	AS‐ArLD		
	Average	Average	*p*‐value	Average	Average	*p*‐value	
Age	41.8 ± 13.3	54.6 ± 16.4	0.21	56.4 ± 12.42	59 ± 15.12	0.74
C‐reactive protein (mg/dl)	1.2 ± 0.9	1.8 ± 2.8	0.59	0.2 ± 0.4	0.08 ± 0.2	1.0	
Creatinine (mg/dl)	0.68 ± 0.13	0.70 ± 0.08	0.73	0.85 ± 0.14	0.75 ± 0.12	0.24
Glucose (mg/dl)	96.4 ± 16.8	107.4 ± 23.7	0.45	104 ± 18.0	75.6 ± 29.0	0.095	
HbA1c (%)	5.62 ± 0.40	5.87 ± 0.64	0.60	5.16 ± 0.60	5.18 ± 0.77	0.98
AST (UI/l)	30.0 ± 12.9	65.8 ± 62.7	0.30	49.4 ± 24.8	74.6 ± 43.2	0.42	
ALT (UI/l)	48.8 ± 29.7	42.4 ± 11.0	1.0	48.0 ± 24.2	41.6 ± 22.8	1.0
**AST/ALT**	0.7 ± 0.2	1.4 ± 1.1	0.17	**1.1 ± 0.4**	**1.8 ± 0.5**	**0.031**	
GGT (UI/l)	27.0 ± 12.8	104.8 ± 124.1	0.17	355.2 ± 481.2	239 ± 236.2	1.0
BILI total (mg/dl)	0.6 ± 0.1	1.3 ± 1.8	0.94	1.4 ± 1.4	1.4 ± 0.5	0.33	
BILI direct (mg/dl)	0.22 ± 0.04	0.88 ± 1.52	0.88	0.72 ± 1.06	0.56 ± 0.31	0.30
Alkaline phosphatase (UI/l)	76.8 ± 15.8	128.8 ± 80.7	0.17	105.8 ± 58.4	126.2 ± 32.5	0.23	
Total protein (mg/dl)	73.2 ± 3.1	72.6 ± 3.9	0.89	66.0 ± 5.2	73.2 ± 7.1	0.056
Albumin (g/l)	43.4 ± 2.7	41 ± 3.5	0.41	41.8 ± 4.9	41.6 ± 3.7	0.68	
Sodium (mEq/l)	141.4 ± 2.7	143.0 ± 2.0	0.34	140 ± 2.1	141.4 ± 0.8	0.17
Potassium (mEq/l)	4.6 ± 0.6	4.6 ± 0.8	0.98	3.9 ± 0.3	4.12 ± 0.3	0.51	
Leukocytes	7842 ± 1871	6568 ± 1118	0.25	6352 ± 1058	4552 ± 1546	0.15
Hemoglobin (g/l)	141.8 ± 19.8	146.2 ± 29.6	0.46	137.6 ± 30.5	134.6 ± 15.2	0.80	
MCV	90.1 ± 7.1	101.4 ± 9.4	0.06	99.6 ± 12.3	99.7 ± 5.3	0.55
**Platelets**	286,800 ± 98,519	226,800 ± 89,340	0.34	**173,800 ± 21,913**	**90,600 ± 24,203**	**0.0079**	
**Prothrombin time (%)**	94.0 ± 8.5	86.0 ± 18.7	0.68	**93.8 ± 8.8**	**73 ± 15.5**	**0.047**
**INR**	0.98 ± 0.08	1.08 ± 0.19	0.53	**1.02 ± 0.08**	**1.22 ± 0.13**	**0.047**	
MELD	6.2 ± 0.4	8.2 ± 4.3	0.72	7.6 ± 2.1	9.4 ± 2.1	0.32
CHILD PUG	5.00 ± 0.01	6.20 ± 2.68	0.99	5.60 ± 1.34	5.60 ± 0.89	0.99	
Total cholesterol (mg/dl)	173.4 ± 62.4	203.8 ± 24.7	0.46	250.8 ± 126.9	168.8 ± 29.1	0.15
Triglycerides (mg/dl)	154.0 ± 120.4	119.2 ± 31.5	1.0	270.6 ± 286.6	235.2 ± 326.0	1.0	
**Ammonium (μM)**	**90.1 ± 19.1**	**220.9 ± 40.2**	**0.0005**	**86.8 ± 23.4**	**193.9 ± 30.8**	**<0.0001**

Abbreviations: ALT, alanine aminotransferase; AS‐ArLD, advanced‐stage alcohol‐related liver disease; AS‐NAFLD, advanced‐stage nonalcoholic fatty liver disease; AST, aspartate transaminase; BILI, bilirubin; CHILD PUG, Child–Pugh score; GGT, gamma‐glutamyl transferase; INR, international normalized ratio; IS‐ArLD, initial‐stage alcohol‐related liver disease; IS‐NAFLD, initial‐stage nonalcoholic fatty liver disease; MCV, mean corpuscular volume; MELD, model for endstage liver disease.

### 3.3 Correlation between ammonium concentration and clinical biomarkers of fatty liver disease

No statistically significant difference was found for any of the blood parameters assessed between initial and advanced NAFLD (Table 1) other than for ammonium. By contrast, there were statistically significant differences between initial and advanced stages of ArLD in the ratio between aspartate transaminase and alanine aminotransferase (AST/ALT ratio), platelets, prothrombin time, and international normalized ratio (INR) (Table 1).

**FIGURE 5:**
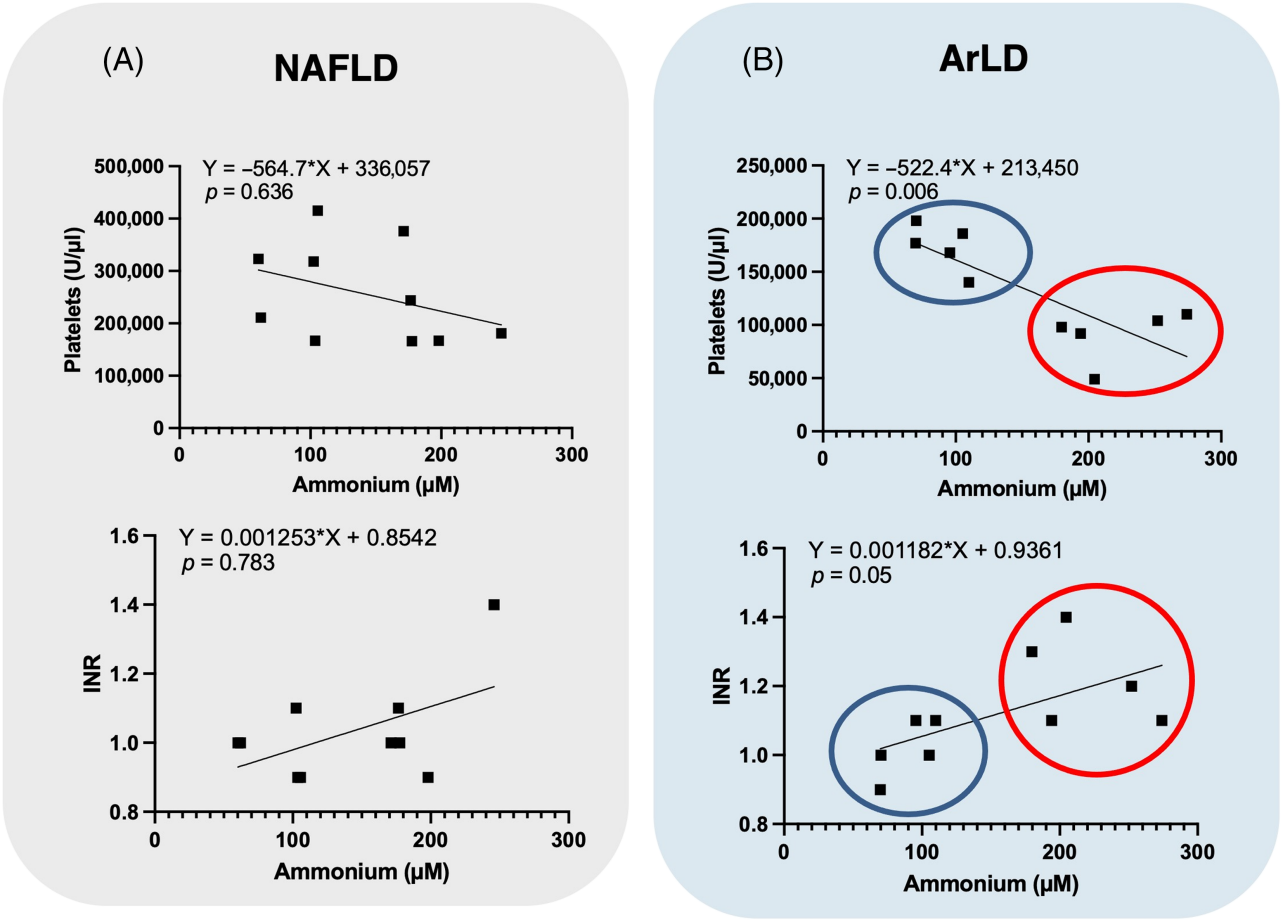
Nonparametric Spearman correlations between platelets and ammonium concentration (top) and international normalized ratio (INR) and ammonium concentration (bottom) for patients with liver disease from either (A) Nonalcoholic fatty liver disease (NAFLD; n = 10) or (B) Alcohol‐related liver disease (ArLD; n = 10). The blue and red circles show the two data groups corresponding to initial and advanced stages of alcoholic fatty liver disease

There was a significant negative correlation between platelets and plasma ammonium concentration in patients with ArLD but not in patients with NAFLD (Figure 5, Table S4). A borderline significant positive correlation was found between INR and ammonium concentration (*p* = 0.054) in ArLD patients. The ArLD data were clustered in two nonoverlapping groups for the initial and advanced stage of the disease (blue and red circles, respectively; Figure 5B).

## 4 DISCUSSION

Blood endogenous ammonium concentrations above 100 μM are strongly associated with severe liver diseases, such as hepatic encephalopathy, NASH, and hepatitis C.^15^ However, accurate quantification methods capable of detecting upregulated ammonium concentrations in blood at treatable stages of the disease have not been developed for clinical use. We have developed a robust protocol capable of quantifying ammonium in plasma by ^1^H‐NMR that is also applicable to other biological fluids. The protocol could readily be adopted as a diagnostic screening tool for diseases where ammonia is a biomarker, particularly for liver fibrosis in patients with NAFLD and ArLD, provided that larger cohort studies validate the data presented.

Ammonium is well suited for analysis by ^1^H‐NMR. NH_4_^+^ exhibits heteronuclear scalar spin–spin couplings, which are rarely observed because of quadrupolar line broadening.^23^ When the ^14^N atom is coordinated symmetrically giving a tetrahedral conformation, ^14^N loses its quadrupolar electric moment that results in coupling with the hydrogen atoms^24^ and the signal then corresponds to a triplet with equivalent intensities. However, ammonium quantification is challenging because it is affected by several variables. The present study critically assesses factors influencing accurate NH_4_^+^ quantification, including solvent deuteration, pH, and pulse sequence.

The choice of solvent substantially affects ammonium quantification, and aqueous and organic solvents were examined. In metabolomic and small‐molecule studies by ^1^H‐NMR, D_2_O is generally used as a solvent. However, this solvent cannot be used for ^1^H‐NMR quantification of molecules like ammonium that have labile protons that can exchange with the ^2^H atoms of the solvent molecules, causing partial or total signal suppression of the signal, depending on the ^2^H/^1^H ratio in the solution. Figure 1 shows the effect of 10% D_2_O in solution on the ^1^H‐NMR spectrum, splitting the ammonium signal into a triplet from mono‐deuterated ammonium. The shift in center frequency of the multiplet is due to an isotopic effect caused by the deuterium atom, and the apparent increase in line width is attributed to the ^2^ *J*_HD_ coupling (Figure 1A). To shift the ammonium/ammonia equilibrium to the cation form for accurate detection, acidification of the samples was required. This ensured sufficient protonation of ammonia, thereby excluding confounded quantitation of NH_4_^+^.

At the 10 mM ammonium concentration used to set up this protocol, ammonium quantification was accurate for pH values between 0 and 3. However, as SNR and FWHM increased as pH decreased, the quantification of solutions with low ammonium concentrations would benefit from the higher SNR obtained at the lowest accessible pH. The changes in SNR and FWHM can be explained by the dissociation of the ^1^H atoms of ammonium. In solution, each of the four ^1^H atoms have the same dissociation probability. The dissociation rate constant *k*_*d*_ is highly affected by pH, decreasing as pH acidifies.^24^ At low exchange rates (*k*_*d*_ ≪ *J*_NH_), spectral lines are sharper, resulting in improved SNR. The analysis of biological samples by ^1^H‐NMR is often performed in nonacidic pHs with D_2_O as the choice of solvent, which probably explains why ammonium present in biofluids has not been previously detected by ^1^H‐NMR. We also observed that water presaturation pulse sequences led to a dramatic decrease of the ammonium signal caused by the proton exchange between ammonium and water. To presaturate the water peak, radiofrequency irradiation is applied to water protons that are continuously exchanging with ammonia, resulting in peak suppression for both molecules.

The protocol was therefore optimized using DMSO as a solvent. Because accurate pH determination relies on using a protic solvent, samples were acidified with TFA, the acid being chosen because it has a proton‐free counterion and therefore gives no ^1^H NMR signal. Acidification also assisted the precipitation of the proteins and lipids of the sample. After acidification, the solution was centrifuged and the supernatant was pipetted off for the next stage of the process. A 200‐μl volume of the TFA was sufficient to accurately quantify ammonium concentration in plasma with a RMSE of 1.6 μM, a LOD of 3 μM, and LOQ of 5 μM. The protocol therefore enables precise and reproducible quantification of ammonium concentration far beyond the typical range found in human plasma (from 40 μM in healthy subjects to more than 100 μM in pathological cases). Quantification also confirmed that the yield of the lyophilization procedure was 100%, that is, it did not result in losses of ammonium, and that the addition of TFA to the sample does not interfere with the internal standard for quantification.

Plasma samples from healthy controls and four patient groups were processed using the above protocol to quantify the ammonium concentration. Plasma contains approximately 92% water and 8% solids.^25^ Using a well‐known protocol for protein and macromolecules precipitation, TFA was added to plasma samples to obtain a nearly solids‐free solution after supernatant removal.^26^ The early‐stage ArLD and NAFLD disease groups showed some differences in ammonium levels of around 50 μM variation, where the mean concentration was higher than for the control group. However, this difference between control and early‐stage groups was not statistically significant. The variation of 50 μM may be due to differences in disease severity within the patient group in the initial stage of classification. By contrast, a striking and statistically significant increase in ammonium concentration was observed for each of the two liver disease groups compared with both initial‐stage and control subjects.

An interesting finding of this study is that ammonium concentration is strongly correlated with platelet levels (*p* = 0.075) and with INR, especially for the ArLD group. At advanced stages of chronic liver disease such as liver cirrhosis, patients may develop portal hypertension, which in turn leads to the development of clinical decompensations of cirrhosis. In this case, spleen size may increase, and peripheral blood platelet levels may decrease, as thrombopenia is a marker of portal hypertension in patients with cirrhosis. Also, coagulation tests such as INR are frequently used to stage the platelet synthesis capacity of the liver, which may be impaired by disease and used in liver function scores. The coagulation tests are also used for staging standardized numbers such as model for endstage liver disease (MELD) or Child–Pugh scores (Figure S1). The correlation between the ammonia levels and platelet counts and INR levels in patients with ArLD may therefore be reflecting a correlation with ammonium levels and more advanced stages of liver disease, such as the appearance of portal hypertension or the impairment of liver synthesis capacity.

Although a limitation of this study is the small cohort assayed, it is striking that the ammonium assay protocol is capable of clearly discriminating the samples corresponding to healthy and early‐stage–diseased subjects from those of patients with advanced stages of the disease. Taken together, these data support the notion that there is a direct correlation between blood ammonium concentration and the disease stage of patients with ArLD and NAFLD, and that when validated by larger sets of samples may have clinical application.

In conclusion, we have developed a robust, reliable, and biopsy‐free protocol to quantify ammonium in biological fluids with a wide dynamic range of concentrations, relative to other methods currently available. To demonstrate potential applications within medical diagnostics and disease staging, we quantified the ammonium concentration in blood plasma samples of patients suffering from a steatohepatic liver condition and showed that the method can be used to discern between some stages of the disease retrospectively. Prospective studies with a larger cohort of patients should validate the clinical applicability and, in future, the protocol could be used in a clinical setting either for population screening or to assess treatment efficacy.

## Supporting information


**Table S1:** Ammonium peak characterization in H_2_O/D_2_O (90:10) with pH dependance. The experiments were done using a 14 T Bruker 600 UltraShield TM. The pulse sequence applied was a proton zg90 with the following NMR parameters: acquisition time of 2.5 s, 64 K data points, 90° flip angle, d_1_ = 15 s and128 scans.
**Table S2:** Ammonium peak characterization in DMSO/DMSO‐d6 (50:50) with pH dependance. The experiments were done using a 9.4 T Bruker Avance‐III HD spectrometer equipped with a cryoprobe. The pulse sequence applied was a proton zg90 with the following NMR parameters: acquisition time of 2.5 s, 64 K data points, 90° flip angle, d_1_ = 15 s and128 scans.
**Table S3:** Presaturation effect in ammonium NMR quantification in water/D_2_O samples. The experiments were done using a 14 T Bruker 600 UltraShield TM. The pulse sequence applied was presaturation using noesypr1d with the following NMR parameters: acquisition time of 2.5 s, 64 K data points, 90° flip angle, d_1_ = 15 s,128 scans, and O1d of 7.71 ppm.
**Table S4.** Non‐parametric Spearman correlation using pooled data (initial and advanced) of ammonium as independent variable. In green, the correlations whose p‐value is ≤ 0.05.
**Figure *S*1:** Graphical representation of non‐parametric Spearman correlation between Child‐Plugh score and ammonium (top) and MELD (model for end‐stage liver disease) and ammonium (bottom) for patients with fatty liver disease from either (A) Nonalcoholic fatty liver disease (NAFLD; n = 10) or (B) Alcohol‐related liver disease (ArLD; n = 10).
