# Supplementary material for: Ammonium quantification in human plasma by proton nuclear magnetic resonance for staging of liver fibrosis in alcohol‐related liver disease and nonalcoholic fatty liver disease
Source: NMR Biomed. 2022 May 9;35(9):e4745. doi: 10.1002/nbm.4745 (PMC9541340; doi:10.1002/nbm.4745)
Supplement: Supplementary file 1 — Table S1: Ammonium peak characterization in H2O/D2O (90:10) with pH dependance. The experiments were done using a 14 T Bruker 600 UltraShield TM. The pulse sequence applied was a proton zg90 with the following NMR parameters: acquisition time of 2.5 s, 64 K data points, 90° flip angle, d1 = 15 s and128 scans. Table S2: Ammonium peak characterization in DMSO/DMSO‐d6 (50:50) with pH dependance. The experiments were done using a 9.4 T Bruker Avance‐III HD spectrometer equipped with a cryoprobe. The pulse sequence applied was a proton zg90 with the following NMR parameters: acquisition time of 2.5 s, 64 K data points, 90° flip angle, d1 = 15 s and128 scans. Table S3: Presaturation effect in ammonium NMR quantification in water/D2O samples. The experiments were done using a 14 T Bruker 600 UltraShield TM. The pulse sequence applied was presaturation using noesypr1d with the following NMR parameters: acquisition time of 2.5 s, 64 K data points, 90° flip angle, d1 = 15 s,128 scans, and O1d of 7.71 ppm. Table S4. Non‐parametric Spearman correlation using pooled data (initial and advanced) of ammonium as independent variable. In green, the correlations whose p‐value is ≤ 0.05. Figure S1: Graphical representation of non‐parametric Spearman correlation between Child‐Plugh score and ammonium (top) and MELD (model for end‐stage liver disease) and ammonium (bottom) for patients with fatty liver disease from either (A) Nonalcoholic fatty liver disease (NAFLD; n = 10) or (B) Alcohol‐related liver disease (ArLD; n = 10). [file NBM-35-e4745-s001.docx]

**SUPPLEMENTARY INFORMATION**

Ammonium quantification (AQua) in human plasma by ^1^H-NMR for staging of liver fibrosis in alcohol-related liver disease and non-alcoholic fatty liver disease

Marc Azagra^1^, Elisa Pose^2^, Francesco de Chiara^1^, Martina Perez^2^, Emma Avitabile^2^, Joan-Marc Servitja^3^, Laura Brugnara^3^, Javier Ramon-Azcón^1,4^, Irene Marco-Rius^1^*

^1^Institute for Bioengineering of Catalonia, The Barcelona Institute of Science and Technology, Barcelona, Spain.

^2^Liver Unit, Hospital Clinic, Faculty of Medicine and Health Sciences, University of Barcelona, Barcelona, Catalonia, Spain.

^3^Institut d’investigacions Biomèdiques Agust Pi i Sunyer (IDIBAPS), Centro de Investigación Biomédica en Red de Diabetes y Enfermedades Metabólicas Asociadas (CIBERDEM), Barcelona, Catalonia, Spain.

^4^ICREA-Institució Catalana de Recerca i Estudis Avançats, Barcelona, Spain.

**Ammonium peak characterization in two different solvents:**

**Supplementary Table 1**: Ammonium peak characterization in H_2_O/D_2_O (90:10) with pH dependance. The experiments were done using a 14 T Bruker 600 UltraShield TM. The pulse sequence applied was a proton zg90 with the following NMR parameters: acquisition time of 2.5 s, 64 K data points, 90º flip angle, d_1_ = 15 s and128 scans.

| H_2_O/D_2_O | | | |
| --- | --- | --- | --- |
|  |  |  |  |
| pH | **^1^H NMR [NH_4_^+^]** | **SNR** | **FWHM** |
| 5,51 | - | - | - |
| 4,86 | - | - | - |
| 4,02 | - | - | - |
| 3,52 | 0.010 | 29.2 | 11.5 |
| 3,03 | 0.011 | 71.0 | 5.1 |
| 2,08 | 0.012 | 112.0 | 3.5 |
| 1 | 0.010 | 97.0 | 3.6 |

**Supplementary Table 2:** Ammonium peak characterization in DMSO/DMSO-d6 (50:50) with pH dependance. The experiments were done using a 9.4 T Bruker Avance-III HD spectrometer equipped with a cryoprobe. The pulse sequence applied was a proton zg90 with the following NMR parameters: acquisition time of 2.5 s, 64 K data points, 90º flip angle, d_1_ = 15 s and128 scans.

| DMSO-d_6_ | | | |
| --- | --- | --- | --- |
|  |  |  |  |
| [TFA] (M) | **^1^H NMR [NH_4_^+^]** | **SNR** | **FWHM** |
| 0.15 | 0.13 | 2790.3 | 2.4 |
| 0.75 | 0.12 | 2620.9 | 2.6 |
| 1.3 | 0.13 | 3582.9 | 2.2 |
| 2.6 | 0.13 | 3499.1 | 1.9 |

**Ammonium peak comparison between ^1^H zg90 acquisition and presaturation pulse sequence.**

**Supplementary Table 3:** Presaturation effect in ammonium NMR quantification in water/D_2_O samples. The experiments were done using a 14 T Bruker 600 UltraShield TM. The pulse sequence applied was presaturation using noesypr1d with the following NMR parameters: acquisition time of 2.5 s, 64 K data points, 90º flip angle, d_1_ = 15 s,128 scans, and O1d of 7.71 ppm.

| **pH** | **^1^H NMR**  **[NH_4_^+^] (M)** | **Presaturation**  **[NH_4_^+^] (M)** |
| --- | --- | --- |
| 5,51 | - | - |
| 4,86 | - | - |
| 4,44 | - | - |
| 4,02 | - | - |
| 3,52 | 0.010 | 0.0001 |
| 3,03 | 0.010 | 0.0003 |
| 2,48 | 0.010 | 0.0003 |
| 2,08 | 0.012 | 0.0005 |
| 1,51 | 0.010 | 0.0004 |
| 1 | 0.010 | - |

**Statistical analysis of the biochemical parameters.**

**Supplementary Table 4.** Non-parametric Spearman correlation using pooled data (initial and advanced) of ammonium as independent variable. In green, the correlations whose p-value is ≤ 0.05.


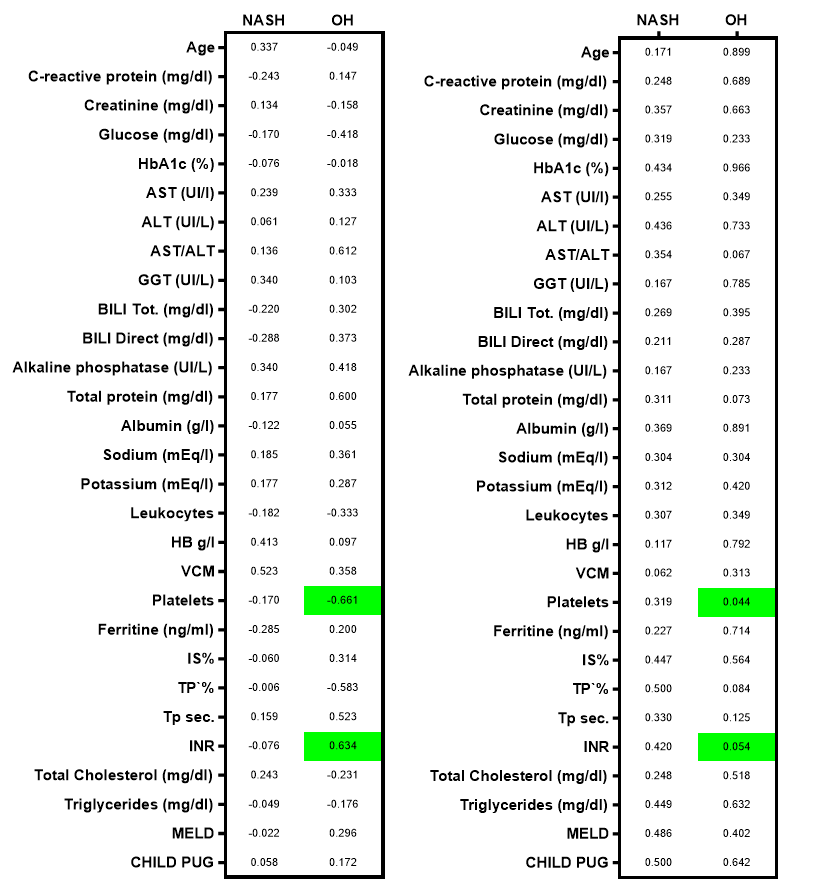


**Spearman correlation**

**Two-tailed p-value**

**Correlation between Child-Pugh score and MELD with ammonium**

**Supplementary Figure 1:** Graphical representation of non-parametric Spearman correlation between Child-Plugh score and ammonium (top) and MELD (model for end-stage liver disease) and ammonium (bottom) for patients with fatty liver disease from either (a) non-alcoholic (n = 10) or (b) alcoholic etiology (n=10).


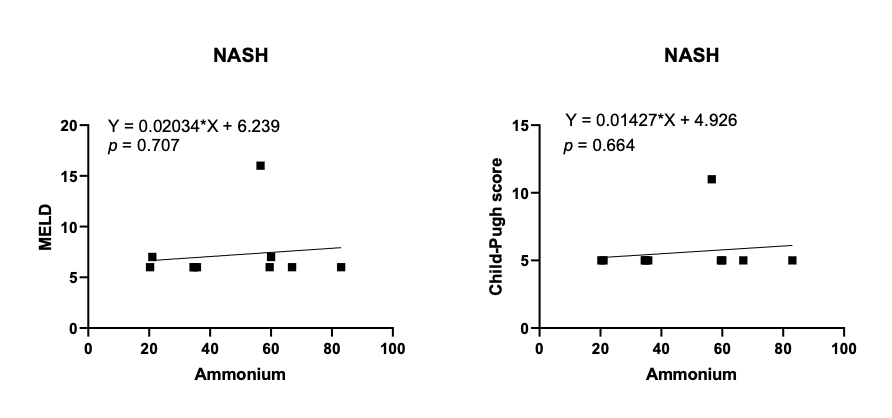

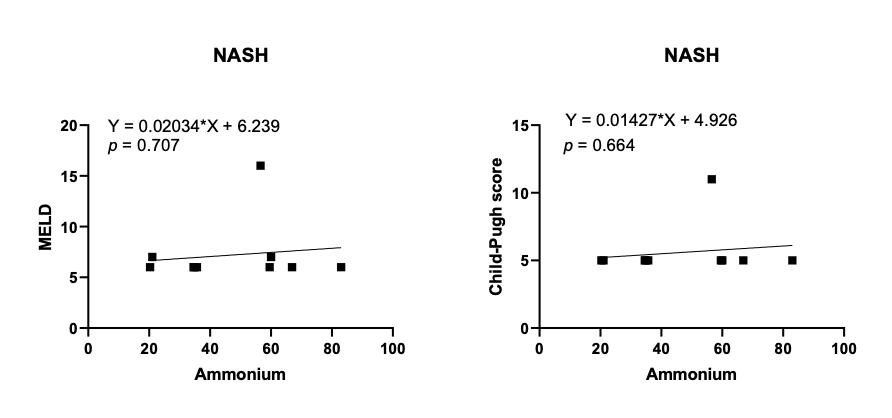


**NAFLD**

**NAFLD**

**(a)**

**(b)**
